# The Lex Coronaria

**Published:** 1885-08

**Authors:** Hooper Alexander

**Affiliations:** Atlanta, Ga.


VOL. II. AUGUST, 1885. NO. 6.

### Original Communications.

### THE LEX CORONARIA.

BY HOOPER ALEXANDER, ESQ., ATLANTA, GA.

"But is that the law?
Ay, marry is't; crowner's quest law."

The editors of THE JOURNAL have asked me to prepare an article for publication which shall set forth the history and status of coroners, both of old time as at common law, and now as by statute, wherein should be mentioned also the matters in which the duty of the coroner impinges upon the profession of medicine. No apology, therefore, is necessary from me. What interest the medical fraternity has in the matter, THE JOURNAL assumes the responsibility of determining. Natheless, let me say, gentlemen of the profession, that there is no occasion for you to affect contempt for him of whom I write. Whatever may be his present condition, he is a gentleman of long descent and has seen better days. He may be in tatters now, but time was when he might brave it with the best dressed one of them all. Long before physicians had ceased to be charlatans, or surgeons were differentiated from barbers, the coroner was a gentleman of credit and renown—nay more, a knight. And Chaucer thought it well to eulogize one as having held the office:

> "At sessions ther was he lord and sire,
> Full often time he was knight of the shire;
> A shereve hadde he ben, and a coronour
> Was no wher swiche a worthy vavasour."

Although his name, being of evident Latin origin, might lead one to think the office at least of later date than the conquest, yet it is certain that coroners existed in Saxon times. Probably the true form of the title is that by which he is still known among the rural population of England. Every one will remember the grave-digger's crushing answer—"I tell thee the *crowner* hath sat on her and finds it Christian burial, therefore, make her grave straight." Most authorities make his origin coeval with the sheriff's, and he, at least, is "genuine Saxon, by the soul of Hengist." Certain it is that when the government of the shires passed from the control of the earls into the hands of the sheriffs, the coroner held a share of it. He was by law a magistrate exercising some high judicial functions, a conservator of the peace, no sinecure in those troublous times.

At common law, none might be a coroner under the degree of a knight, and as far back as the days of Edward I. it was enacted by statute,

"That through all shires sufficient men shall be chosen to be coroners of the most loyal and wise knights," etc.

Later, it seems to have been sufficient that they should own land enough for knighthood, without actually being knights, and later still, that they own land enough "whereof he may answer to all manner of people." Blackstone, who wrote in the last century, complains that "now, through the culpable neglect of gentlemen of fortune, this office has been permitted to fall into disrepute and get into low and indigent hands." The reason for requiring a property qualification seems to have been that they might be responsible in fines and damages for maladministration of official power. And, by the laws of Edward III., if a county elected a coroner not thus qualified, the county was responsible for his fines. Poverty was good ground for removal. One writer speaks in deprecatory manner of there having been instances in which men were elected coroners so illy fitted that they had been removed and had "entered on the trade of a common merchant." In Georgia the slight danger of improper exercise of power is met by requiring a bond of five hundred dollars. When the coroner acts as sheriff, the ordinary may require additional bond.

Like most Saxon offices, that of coroner is close to the people. It has always been elective. True, the Lord Chief Justice of England is a sort of *ex-officio* coroner, and there are others whose appointment is vested in some corporation or attached to some franchise. A curious office of this sort was that of coroner of the verge, a person appointed by the Lord Steward of the Household, who had peculiar jurisdiction within the verge, that is, within a radius of twelve miles around the place where the king slept. But the coroner proper—the true progenitor of the Georgia coroner—was always elected by the people "in full county," that is, at the meeting of the county court. As a consequence of this mode of choice, the office never did determine with the demise of the king like other English offices, but was held for life by one once chosen. He could be removed either by being chosen sheriff, or for cause, by judicial proceedings. Among the grounds enumerated as cause for removal, one is poverty. In Georgia, coroners are elected by the people for a term of two years. In cases of vacancy or failure to elect, the ordinary may appoint one till the vacancy can be lawfully filled.

The powers and duties of coroners were formerly quite numerous. Of course, his principal function always was, and still is, in the matter of inquisitions of deaths. In England this duty carried with it extensive powers. At common law he was to pronounce the judgment of outlawry. He also inquired into all matters pertaining to the taking of royal fish, the finding of treasure, the forfeiting of deodands, etc. In the matter of treasure troves, it was directed by statute that he should inquire "who were the finders, and likewise who is suspected thereof, and that may be well perceived, where one liveth riotously, haunting taverns, and hath done so of long time, hereupon he may be attached for suspicion." A pretty broad discretion. His ministerial functions were the same at common law as in Georgia, where, aside from his duty in the matter of inquests, he is, under certain circumstances, the sheriff's substitute. With us, he is the keeper of the jail when the sheriff is imprisoned, or absent from the county leaving no deputy; rules against sheriffs are properly executed and returned by the coroner; in actions at law, when the sheriff is a party to the cause, the process is directed to the coroner and to the sheriffs of the adjoining counties. Whenever the sheriff is disqualified and it does not appear on the face of the proceedings, or, if he refuses to perform a duty, upon affidavit of the facts being made by any one, the clerk may deliver the process to the coroner and compel him to return it.

At common law coroners had no fees, and the statute of Westminster affirmed this "on pain of great forfeiture to the king." In the reign of Henry VII. they were allowed 13s. 4d. in the inquisitions of murder, of the goods and chattels of the slayer, if he have any goods, and, if he have no goods, "of such amercements as shall fortune any township to be amerced for the escape of the murderer." By a statute of the succeeding reign it was a serious penalty for a coroner to demand this fee in cases of death by accident. These laws were re-affirmed in the reign of George II. In Georgia the coroner is entitled to the sheriff's fees when discharging the duties of that officer. He is also allowed ten dollars for each inquest and fifteen dollars for furnishing the coffin and burial expenses, provided that he shall not receive more than fifteen hundred dollars per year out of the county treasury on these two accounts.

In the matter of inquests over dead bodies, the old English statutes were very minute; that of Edward III., in particular, giving very detailed instructions. The limits of this article forbid my going into them, and I confine myself to the proceedings in Georgia.

In this State it is the coroner's duty to hold inquests: 1. Of all violent, sudden, or casual deaths. 2. Of all deaths in prison without an attending physician. 3. Of all dead bodies found, whether of persons known or unknown. 4. Of all dead bodies of persons who have died or disappeared under suspicious circumstances. 5. Of the dead bodies of persons of whom affidavit may be made that they came to their death by violence or foul play. 6. Whenever ordered by a court having criminal jurisdiction. In interpreting the word "sudden" in the first of these classes, the coroner generally gives a very liberal construction. It does not, however, properly or lawfully extend to cases of death by accident or the act of God, where there are witnesses and no reason to suspect foul play. And as to casual death, it has been held by the Supreme Court, in a case of death by railroad accident, that the coroner had no vested right to hold an inquest and collect his fees. Where death occurs from violence in the presence of witnesses, and the person accused is arrested and undergoes examination, no inquest is necessary.

Whenever the proper circumstances for holding an inquest are brought to the coroner's notice, it is his duty to direct a precept to some constable, commanding him to summon eighteen good and lawful men of the county to the spot. This precept must be executed by the constable, but if none is to be had, the coroner may summon the jury himself, in which case he is probably entitled to the constable's fee of one dollar. Defaulting jurymen may be fined ten dollars by the coroner, who may issue execution to collect the fine. The defaulter may file an excuse with the ordinary, who may pass thereon at the next term of his court. No provision is made for paying the jurors. In a city or town the jury is composed of twelve men, of whom seven may bring in a verdict. Elsewhere six is a sufficient number, of whom a majority may make a finding.

The coroner administers to the jurymen an oath to "diligently inquire and true presentment make on behalf of the State of Georgia, how and in what manner C. D. (or the person deceased unknown), now here lying dead, came to his death, and of such other matters relating to the same as shall be lawfully required of you according to evidence." It is his duty then to instruct the jury. The substance to this charge is, that they shall inquire how the person died, and if by murder or manslaughter, who were the principals and accessories to the crime; of the means employed and all the presumable circumstances; if by misfortune or accident, how, and who were present; whence the person came, who were his parents, relations and neighbors, and the finders of his body; whether he died where the body was found or elsewhere; and, if elsewhere, who moved the body, and all the circumstances. If the deceased died in prison, he is to charge the jury to inquire if it were from hard usage, and, if so, by whom? If it is a suicide, he is to instruct the jury to inquire of the means used and other circumstances.

Under this charge the jury has broad license to institute a full and unrestricted inquiry, and to this end the coroner may subpœna witnesses, compel their attendance and swear them, and they are liable to the penalties of perjury, the proceeding being in its nature judicial. If the inquest disclose facts which may lead to a prosecution for homicide, the coroner may recognize witnesses to appear and testify in the Superior Court.

After a full inquiry into all the facts and circumstances, the jury make up their verdict, which ought, of course, to contain a full response to the charge as far as possible from the evidence. The coroner must commit the substance of the testimony to writing and return this with all the other papers to the next Superior Court.

As the inquest must be held upon a view of the body, the coroner has power to disinter those buried, and may, if necessary, raise the *posse comitatus* to do this. But a severe penalty is annexed to any disinterment out of malice or mischief or without good ground, the coroner being liable if he acts upon his own motion, or if upon affidavit, then the person swearing.

The effect of the finding is very much modified with us from the common law. In England an indictment might be thereon directly predicated when the jury brought in a verdict that any person was guilty of murder, and the time was when an acquittal would not be allowed against the verdict. By the Constitution of the United States "no person shall be held to answer for a capital offense but upon presentment or indictment by the grand jury."

So that now the principal use of the inquest is to get up all the facts and circumstances on the spot, while the act is still fresh, and the witnesses may give detailed accounts of everything which may tend to throw light upon the occurrence. The record made fixes the testimony, and may afford valuable evidence on the trial of the slayer. It is also data from which the grand jury may find their bill. When any one is pointed out by the evidence or verdict as suspected of homicide, the coroner may issue a warrant for his arrest, to be returned to some preliminary court.

Of course, the testimony of a medical practitioner is nearly always necessary. His evidence is generally of a *quasi* expert nature, and may be altogether based upon a hypothetical statement of fact. Generally, however, he examines the body. It is not within the province of this article to lay down any rules for governing him. He must testify in the light of his own experience and knowledge. *Post mortem* examinations can be made only when the coroner and a majority of the jury believe them necessary. By the act of 1880, when this is the case, the coroner may employ a competent physician and make a contract with him. His fee is paid by the county, but may not exceed twenty dollars.

It is a curious little piece of our judicial history to inquire into the law of *post mortems* by a jury of inquest. In 1850 the Legislature passed a very liberal act for remunerating physicians for these services, but the original codifiers, who very often moulded things to suit their own views of the law, modified the statute considerably in the codification, and the adoption of the Code as a whole by the Legislature was held by the Supreme Court to have worked a repeal of the act, except in so far as codified. And so the examining physician lost his fee. The matter was partially remedied by the act of 1880.

Whenever the verdict suggests poison, the coroner may have the viscera and contents of the stomach and intestines examined by a chemist, and the reasonable expenses are to be paid out of the county treasury.

Where an inquest has been held, and one is afterwards put on trial and convicted of the killing, the costs of the proceedings are taxed against him in the final bill of costs.

The coroner's office is one, the utility of which has been much discussed, and it is frequently said that it should be abolished. The wisdom of such a course would be doubtful. There can be no question that the verdict is often silly and of no worth. This may raise the necessity for improvement in the conduct of inquests and the choice of better officers. That the office itself and its duties should be abolished is quite another matter.

The coroner was originally intended as a sort of tribune of the people, and we cannot doubt that, in the early and sanguinary days in England, his power was a serious menace and effective check upon the lawless and tyrannical. The very absence now of the crimes which were his *raison d'etre* in early days is proof of his utility, and it might well be thought bad policy to dispense with the sentinel because the enemy has retired. The same argument which would lead to the abolition of this office would apply as forcibly to other preliminary courts of inquiry. True, the inquisitorial powers of the coroner might be vested in the justice of the peace, who may sometimes discharge these functions now. But this would not diminish the costs, nor is it likely that it would lead to better results. Besides, it would be necessary to make some disposition of his ministerial powers as the sheriff's substitute. The true remedy rests with the people, and must be in the selection of better men; and this, no doubt, will be done whenever occasion requires. The old power of the coroner is latent, but whenever use demands its reappearace, the procreative force of the ballot will beget it upon the body of this barren office.

This is a day and country of innovations, and we, in Georgia, have been very bold to make them, and, in making them, we have often done well. But it was a sage remark of Blackstone's that "it hath been an ancient observation in the laws of England that whenever a standing rule of law, of which the reason, perhaps, could not be remembered or discerned, hath been wantonly broken in upon by statutes and new resolutions, the wisdom of the rule hath in the end appeared from the inconveniences that have followed."

